# Who uses running apps and sports watches? Determinants and consumer profiles of event runners’ usage of running-related smartphone applications and sports watches

**DOI:** 10.1371/journal.pone.0181167

**Published:** 2017-07-21

**Authors:** Mark Janssen, Jeroen Scheerder, Erik Thibaut, Aarnout Brombacher, Steven Vos

**Affiliations:** 1 School of Sports Studies, Fontys University of Applied Sciences, Eindhoven, The Netherlands; 2 Department of Industrial Design, Eindhoven University of Technology, Eindhoven, The Netherlands; 3 Policy in Sports and Physical Activity Research Group, University of Leuven, Leuven, Belgium; INSEP, FRANCE

## Abstract

Individual and unorganized sports with a health-related focus, such as recreational running, have grown extensively in the last decade. Consistent with this development, there has been an exponential increase in the availability and use of electronic monitoring devices such as smartphone applications (apps) and sports watches. These electronic devices could provide support and monitoring for unorganized runners, who have no access to professional trainers and coaches. The purpose of this paper is to gain insight into the characteristics of event runners who use running-related apps and sports watches. This knowledge is useful from research, design, and marketing perspectives to adequately address unorganized runners’ needs, and to support them in healthy and sustainable running through personalized technology. Data used in this study are drawn from the standardized online Eindhoven Running Survey 2014 (ERS14). In total, 2,172 participants in the Half Marathon Eindhoven 2014 completed the questionnaire (a response rate of 40.0%). Binary logistic regressions were used to analyze the impact of socio-demographic variables, running-related variables, and psychographic characteristics on the use of running-related apps and sports watches. Next, consumer profiles were identified. The results indicate that the use of monitoring devices is affected by socio-demographics as well as sports-related and psychographic variables, and this relationship depends on the type of monitoring device. Therefore, distinctive consumer profiles have been developed to provide a tool for designers and manufacturers of electronic running-related devices to better target (unorganized) runners’ needs through personalized and differentiated approaches. Apps are more likely to be used by younger, less experienced and involved runners. Hence, apps have the potential to target this group of novice, less trained, and unorganized runners. In contrast, sports watches are more likely to be used by a different group of runners, older and more experienced runners with higher involvement. Although apps and sports watches may potentially promote and stimulate sports participation, these electronic devices do require a more differentiated approach to target specific needs of runners. Considerable efforts in terms of personalization and tailoring have to be made to develop the full potential of these electronic devices as drivers for healthy and sustainable sports participation.

## Introduction

This paper focuses on event runners’ usage of running-related smartphone applications (apps) and sports watches. Running is one of the most popular forms of sports participation in Western Europe. Currently, in the EU-28, there are approximately 50 million running participants. This is almost 10% of the total EU-28 population [1]. In the US, approximately 42 million people (of a total population of 323 million citizens) partake in running [2]. The running boom is consistent with a more general development toward more recreational, unorganized, and lighter forms of sports [1,3]. Some of the interesting qualities of running are its health-related focus, imposes hardly any restrictions on age, requires no specific infrastructure, and can be practiced independently of time and place [4,5]. Running attracts diverse participants in terms of socio-demographic characteristics such as age, sex, and socio-economic status [6,7], but also in terms of motives (e.g., health, freedom, social experience, fun, and performance enhancement) [8,9] and experience (e.g. both novice and experienced runners).

Moreover, there has been a shift from running in private track and field clubs to large numbers of people running individually or in small groups. The ‘new’ carrier of the growing popularity of recreational and unorganized running are running events [1,6,7,10]

Given the large number of running participants and the rise in heterogeneity, certain challenges need to be tackled. Personalized guidance and support is losing ground, often resulting in drop-out due to injuries or demotivation [11–13]. Substantial guidance is necessary to maintain sensible and sustainable sports participation among novice and less experienced runners [14,15].

In line with the progression toward more unorganized running, in recent years, there has been an exponential increase in the availability and use of sports and physical activity-related monitoring devices [16–20]. According to the intended usage, the two different groups of monitoring devices in sports can be classified as: (i) sports watches/wearable devices, which are specifically designed for sports; and (ii) apps specifically designed for sports [15]. These apps turn a smartphone, which can be seen as a non-specific sporting good, into a sport-related good [17].

In 2015 in the US, 82% of the adult population (18–49 years of age) owned a smartphone, whereas 15% of this population owned an activity tracker [21]. An average American smartphone carries 22 downloaded apps, and 30% of them are sports-related (including both active participation and media sports) [22]. Roughly 20% of smartphone users utilize health-related apps [23], and about 3% of all available apps in the app stores are health-related apps capable of monitoring sports related physical activities. [24].

The increase in user-friendly, low-cost, mainstream technology related to sports is consistent with more general trends such as mHealth [25] (i.e., the use of mobile computing and communication technologies in health care and public health) and Quantified Self [26] (i.e., self-monitoring health outcomes). Indeed, sports and physical activity-related monitoring devices have the potential to contribute to a healthier lifestyle and can become an important driver of behavioral change towards a healthier lifestyle [27]. As such, these electronic devices could also play a role in supporting and monitoring the large group of unorganized runners, who lack professional training and coaching.

Knowing who uses these types of technology is relevant to adequately address (unorganized) runners’ needs, and to support them in healthy and sustainable running through personalized technology. This study aims to gain insight into the characteristics of (event) runners who use apps and sports watches. The determinants of event runners’ usage of apps and sports watches are identified following a heterodox-based approach [3,28–30] with the incorporation of socio-demographic variables, running-related variables, and psychographic characteristics.

The scope of this paper is on the actual usage of apps and sports watches. However, studies dealing with the usage of sporting goods are scarce [31]. Yet, literature on sports participation and expenditures on sports is widely available (for an overview see e.g. [32–34]). Within this literature, most empirical studies have focused on the determining factors of overall sports expenditures [32,35–37]. Only a small number of studies have analyzed specific sports expenditure categories [30]. As a consequence, little is known about which determinants influence expenditures on wearable sports monitoring devices. However, given the supposed direct relationship between expenditure on sporting goods and usage of sporting goods, an overview is provided of empirical findings of consumer segmentation to detect relevant variables related to sports expenditure and usage. These studies have different theoretical perspectives (for an overview, see [38]).

Several socio-demographic (and socio-economic) variables have been found to be useful in explaining sports consumption behavior. First, the relationship between sporting goods expenditure and age are divergent. In some studies, the youngest groups have been found to spend more [39], while others found that age has a positive or U-shaped relationship with sporting goods expenditure [40]. Lera-López and Rapún-Gárate [35,36] did not find any significant relationship between the two factors. Second, men spend more money on sports than do women [39,41]. Finally, income [32,42–44] and education [35,36,42] are found to be positively related to sports expenditure. Groups with lower education levels seem to spend less on sporting goods, while people with a higher level of education are more likely to spend more money on sports. The results presented above indicate that demographic variables such as gender, age, income, and education are useful in order to understand sports consumption behavior.

Ohl and Taks [31] have stated that sports consumption behavior also depends on underlying motivations, such as behavioral variables (see also [45]). Sports-related variables, such as the training frequency and complexity of participation, can be considered as behavioral characteristics [46]. For example, Wicker et al. [47] covered the complexity of participation by performance level, expenditure, intensity of training, time of practice, event participation, years of practice, and organizational context (individual, group, or club). Similar sports-related variables have been used in previous research on triathlons [48,49] as well as in research by Scheerder et al. [3], who argue that these behavioral characteristics seem to be better predictors of sports consumption than demographic variables.

This finding also applies to running. For example, McGehee et al. [50] found that frequency of running, event participation and expenditure on running-related products and services increased in individuals with high levels of running involvement. Moreover, Ogles and Masters [51] found that different types of marathon runners are distinguishable not only by their demographic characteristics but also by their behavioral variables. The abovementioned results indicate that behavioral characteristics are useful in understanding consumer behavior.

Next to socio-demographic variables and behavioral characteristics, psychographic variables (the consumer’s state of mind) determine sports participation and expenditure. These variables provide information about the attitudes, interests, and opinions (AIO’s) that steer consumer behavior [46,52]. Examples used in the literature are the consumer’s personality, lifestyle, values, attitudes, beliefs, motivations, and needs [45,49,53–58]. Although previous research has demonstrated that AIO’s contribute significantly to explaining sports consumption [3], they are less easily obtained compared to the abovementioned socio-demographic, socio-economic, and sports-related variables (i.e. behavioral characteristics). Applied to running, Ogles and Masters [51] found that psychographics can be used to characterize different clusters of runners. Various studies [10,51,59] used similar motives (e.g. health, personal goal achievement, social aspects of running, addiction, competition, and ease of practice) to cluster runners. Although the design of the studies of Ogles and Masters [51], Rohme et al. [59], and Vos et al. [10] are all different, they conclude that adding psychographic variables enriches running profiles.

## Methods

### Data

The research conducted was in line with the ethical principles of the Declaration of Helsinki and the American Psychological Association [60]. The privacy of all participants was guaranteed, and all data was anonymized before analysis. The data used in this study were drawn from the Eindhoven Running Survey 2014 (ERS14). This standardized online questionnaire was developed to collect information among event runners. The questions were based on the Leuven Running Survey 2009 [61] and adapted to event runners. The questionnaire consisted of four sections: (i) the use and interest in running-related apps and sports watches; (ii) socio-demographic characteristics (such as age, gender, and level of education); (iii) running characteristics (such as training frequency, organizational context, main sports, and event participation); and (iv) psychographic characteristics (such as motives and attitudes toward running). For this paper, a sub-dataset (see S1 File) was constructed containing only those runners that participated in the Half Marathon Eindhoven 2014 (21.1k). This distance was selected because of the heterogeneity of the participants, including both experienced and less experienced runners. Data were collected in October 2014. Participants agreed upon registration to be contacted for research or other purposes. All participants that finished the race received an email with an introductory letter (mail text) and a web link to the online questionnaire. The introduction letter informed them about the purpose of the study and the anonymization of the data. In total, 2,172 participants completed the questionnaire (a response rate of 40.0%). The average age of the respondents in the dataset was 41.5 years, ranging from 16 years to 76 years old. Thirty percent of the participants were women, and more than four out of five (86.2%) participants were employed. The socio-demographic backgrounds of the respondents were comparable to other running samples in previous large-scale running studies in Western Europe (for an overview of these studies, see [1]).

### Measurements

#### Dependent variables

The dependent variables in this study included: (i) the use of apps (binary coded as 1 yes / 0 no); and (ii) the use of sports watches (binary coded as 1 yes / 0 no). Respondents were asked to fill in their usage of apps and sports watches (defined as using one or more running-related app(s) / sports watch(es) in the last twelve months). The respondents were also asked to give details about the specific brand and model they used.

#### Independent variables

In line with previous studies on sports expenditures [3,30], a so-called heterodox economic approach was used. Heterodox economic theory assumes that behavior not only depends on the income and the price of the good, but that variables such as subjective feelings and social interactions are more important in explaining human behavior (such as sports consumption). In line with this approach, the set of independent variables included three groups of variables: (i) socio-demographic variables; (ii) running-related variables; and (iii) psychographic characteristics.

The socio-demographic characteristics included gender, age, and level of education. The group of running-related characteristics consisted of variables that are directly related to running and which define the level of running involvement: training frequency (number of runs per week); organizational context (individually, with friends, colleagues and/or running groups or clubs); event participation (total number of running events participated in during last 12 months); and the most practiced sports (running/other sports) were questioned. Table 1 gives the descriptive statistics of the sample for the dependent and independent variables.

**Table 1:** Overview, measurements, and descriptive statistics of the dependent and independent variables.

Variable	Measurement	n	%
App use	Yes	1,091	54.90
	No	897	45.10
Sports watch use	Yes	1,177	60.50
	No	768	29.50
Gender	Male	1,500	77.40
	Female	437	22.60
Age	≤ 35 year	712	37.10
	36–45 year	526	27.40
	≥ 46 year	679	35.40
Education	Lower or middle education	604	31.10
	Higher education	1,341	68.90
Training frequency	≤ 1x/week	536	26.90
	2x/week	859	43.10
	≥ 3x/week	599	30.00
Organizational Context	Individual	1,129	57.60
	Friends, colleagues, small groups	440	22.50
	Clubs	390	19.90
Main sport	Main sport	1,496	75.10
	Not as a main sport	497	24.90
Event participation	1x/year	449	22.50
	2-4x/year	980	49.10
	≥5x/year	565	28.30

To complete the heterodox approach, psychographic characteristics were operationalized and scales were constructed. Respondents were asked to what extent they agreed with 19 items on a five-point Likert scale (ranging from 1 (totally disagree) to 5 (totally agree)). These items were based on previous research [10,51,59] and included in the Principal Component Analysis (PCA) with orthogonal Varimax Rotation (EVA = 55.0%). From this PCA analysis, the items were grouped into four psychographic components, namely (i) running as a sport that is easy to practice (e.g. I can practice running anytime, anywhere), (ii) perceived advantages of running (e.g. Running gives me energy or running is good for my health), (iii) individual motives for quitting (e.g. I would quit running if I get injured or if my spare time decreases), and (iv) social motives for quitting (e.g. I would quit running if my trainer stops or if my running friends stop). Cronbach’s Alpha scores were calculated for each component, and 5 items were removed to increase Cronbach’s Alpha scores, resulting in a total of 14 items used for further analysis. Table 2 gives an overview of these components (i.e. scales), including average score (ranging from 1 to 5), Cronbach’s Alpha’s, and the number of items.

**Table 2:** Overview and descriptive statistics of the psychographic characteristics.

Attitudes toward running	Items	Cronbach’s alpha	n	Mean	Standard Deviation
Running as a sport that is easy to practice	3	0.822	1,951	4.23	0.674
Perceived advantages of running	4	0.856	1,950	4.03	0.475
Individual motives for quitting	4	0.704	1,947	3.11	0.790
Social motives for quitting	3	0.925	1,944	1.61	0.708

### Data analysis

First, descriptive statistics were collected to provide an overview of (i) the sample structure and (ii) the use of apps and sports watches. Second, chi-squared tests (*p*<0.05 was considered significant) with post hoc testing (through z-scores and adjusted p-values (Bonferroni-method)) were conducted to examine differences in the usage of apps and sports watches by the selected socio-demographic and running-related variables. For the psychographic characteristics, mean scores with standard deviation were calculated. Third, binary logistic regression analyses (method = enter) determinants of the use of apps and sports watches were identified. The independent variables were divided into three blocks: (i) socio-demographic variables; (ii) running-related variables; and (iii) psychographic characteristics. For both apps and sports watches, three models were estimated: (i) model 1 consists of socio-demographic variables only; (ii) model 2 consists of both socio-demographic variables and running-related variables; and (iii) model 3 consists of socio-demographic variables, running-related variables and psychographic characteristics. Nagelkerke R^2^ was used as a measure of goodness of fit. Values between 0.10 and 0.20 were considered as satisfactory [62]. The different models were tested for multicollinearity, outliers, and leverage points. No problems with the data were found concerning these aspects. Finally, the results of the full models were used to develop consumer profiles for the use of apps and sports watches. This approach was in line with the approach developed by Scheerder et al. [3] to identify sports apparel consumer profiles.

## Results

Results show that more than 8 out of 10 (86.2%) runners (n = 1,995) used at least one monitoring device over the past 12 months. More than half of the participants (54.9%) reported the use of apps, while 60.5% used a sports watch; approximately 1 out of 4 participants used both apps and a sports watch (27.0%). The brand specific analysis revealed that the most popular app among runners is Runkeeper (50.8%), followed by Runtastic (16.0%), and Nike+ Running (11.1%). Garmin was found to be the most popular brand among users of sports watches (43.9%), whereas Polar (27.4%), TomTom, and Nike (both 7.4%) are used less.

The results of the bivariate analyses are presented in Tables 3 and 4. Significant differences (*p*<0.01 or *p*<0.001) for the use of apps were found for gender, age, training frequency, organizational context, main sport, and event participation. No significant difference in the use of apps was found for education (*p* = 0.087). For the usage of sports watches, significant differences (*p*<0.001) were found for the variables age, training frequency, organizational context, main sports and event participation. Gender and education were not significantly different (*p* = 0.703 and *p* = 0.272) for sports watch usage.

**Table 3:** Results of chi-squared test with post hoc testing for event runners’ usage of apps and sports watches for the socio-demographic variables and running-related characteristics, in percentages.

		Use of Apps		Use of Sports Watches	
		%	p-value	%	p-value
Gender	Male	53.5	*p*<0.001	60.7	*p* = 0.703
	Female	62.5		59.7	
Age	≤ 35 year	66.1^a^	*p*<0.001 ^a-c, b-c^	51.9^a^	*p*<0.001 ^a-b, a-c^
	36–45 year	63.4^b^		62.6^b^	
	≥ 46 year	38.4^c^		67.9^c^	
Education	Lower or middle education	52.5	*p* = 0.087	62.4	*p* = 0.272
	Higher education	56.7		59.7	
Training frequency	≤ 1x/week	64.1^a^	*p*<0.001^a-c, b-c^	39.0^a^	*p*<0.001^a-b, a-c, b-c^
	2x/week	57.8^b^		61.2^b^	
	≥ 3x/week	42.5^c^		78.5^c^	
Organizational Context	Individual	60.6^a^	*p*<0.001^a-c, b-c^	55.2^a^	*p*<0.001 ^a-c, b-c^
	Friends, colleagues, small groups	56.6^b^		58.4^b^	
	Clubs	35.5^c^		76.6^c^	
Main sport	Main sport	50.9	*p*<0.001	64.8	*p*<0.001
	Not as a main sport	66.7		47.2	
Event participation	1x/year	65.5^a^	*p*<0.001 ^a-b, a-c, b-c^	44.4^a^	*p*<0.001 ^a-b, a-c, b-c^
	2-4x/year	57.5^b^		58.7^b^	
	≥5x/year	41.8^c^		76.2^c^	

**Table 4:** Overview of mean scores (and standard deviation) for event runners’ usage of apps and sports watches for the psychographic variables.

Attitudes toward running	Use of Apps	Use Sports Watches
Running as a sport that is easy to practice	4.26 (0.644)	4.25 (0.666)
Perceived advantages of running	4.00 (0.462)	4.09 (0.465)
Individual motives for quitting	3.23 (0.800)	3.06 (0.787)
Social motives for quitting	1.63 (0.712)	1.59 (0.692)

### Running-related apps

Table 5 presents the results of the three binary logistic regression models for the use of apps. Model 1 shows that the use of apps is determined by age. Runners aged 46 years or older were less likely (OR = 0.313, *p*<0.001) to use apps than younger runners (≤ 35 years). No effect was found for gender and education. The second model (Model 2) included both socio-demographic variables and running-related variables. Results show that age remains a determinant variable to predict usage. Older runners (46 years or older) were less likely (OR = 0.424, *p*<0.001) than people in their twenties or thirties to use an app. Running characteristics also significantly contribute to the use of apps. Significant effects were found for organizational context, event participation, and running as a main sport. Runners who run in a club are less likely (OR = 0.584, *p*<0.001) to use an app than individual runners. Also, runners who more frequently participate in events (2–4 times a year: OR = 0.757, *p*<0.05 / ≥5 times a year: OR = 0.545, *p*<0.001) are less likely to use apps than those who run only in a single event each year. The ‘main sport’ variable also determines the usage of apps. Runners who do not consider running as their main sport are more likely to use apps (OR = 1.434, *p*<0.001) than those who consider running as their most important (or only) sport. The training frequency has no significant contribution to the usage of apps.

**Table 5:** Results of the binary logistic regression analysis for event runners’ usage of apps, in odds ratios (Exp (β)) with regards to the reference group (ref.).

		Use of apps		
		Model 1	Model 2	Model 3
Constant		1.958***	2.522***	1.111
Gender	Male	Ref.	Ref.	Ref.
	Female	1.046	1.171	1.147
Age	≤ 35 year	Ref.	Ref.	Ref.
	36–45 year	0.908	1.090	1.123
	≥ 46 year	0.313***	0.424***	0.449***
Education	Lower or middle education	Ref.	Ref.	Ref.
	Higher education	0.964	0.884	0.860
Training frequency	≤ 1x/week		Ref.	Ref.
	2x/week		1.103	1.128
	≥ 3x/week		0.792	0.855
Organizational context	Individual		Ref.	Ref.
	Friends, colleagues, small groups		0.919	0.899
	Clubs		0.584***	0.556***
Main sport	Main sport		Ref.	Ref.
	Not as a main sport		1.434**	1.402*
Event participation	1x/year		Ref.	Ref.
	2-4x/year		0.757*	0.744*
	≥5x/year		0.545***	0.545***
Attitudes toward running	Running as a sport that is easy to practice			0.971
	Perceived advantages of running			1.071
	Individual motives for quitting			1.205**
	Social motives for quitting			1.050
Nagelkerke R^2^		0.090	0.144	0.149

* = *p*<0.05

** = *p*<0.01

*** = *p*<0.001

The full model (Model 3) shows that the three selected blocks of variables contribute to the explanation of the usage of apps. Age, organizational context, main sport, event participation, and individual motives for quitting running are associated with app usage. Runners aged 46 years or older were less likely (OR = 0.449, *p*<0.001) to use apps than runners 35 years or younger. On the other hand, people whose main sport is not running are more likely (OR = 1.402, *p*<0.001) to use apps. Club runners are less likely (OR = 0.556, *p*<0.001) to use an app, as are runners who have participated in two or more events than those who run individually or participate in only one event per year. With regard to attitudes and motives toward running, runners who score higher on individual motives for quitting are more likely (OR = 1.205, *p*<0.001) to use apps.

### Sports watches

The results of the three binary logistic regression models for sports watches are shown in Table 6. Model 1 consists only of socio-demographic variables. Model 2 is a more extended model composed of socio-demographic variables and running-related variables. Model 3 is the most extended model, showing a heterodox set of variables: socio-demographic variables, running-related variables, and psychographic characteristics. In Model 1, in line with the results for the use of apps, only age has a significant contribution to the use of sports watches. However, in contrast with the use of apps, runners aged 46 years or older (OR = 1.125, *p*<0.001) and runners between the ages of 36 and 45 years (OR = 1.593, *p*<0.001) are more likely to use sports watches than event runners aged 35 years or younger. No significant effect was found between age and education.

**Table 6:** Results of the binary logistic regression analysis for event runners’ usage of sports watches, in odds ratios (Exp (β)) with regards to the reference group (ref.).

		Use Sports Watches		
		Model 1	Model 2	Model 3
Constant		0.954	0.384***	0.133***
Gender	Male	Ref.	Ref.	Ref.
	Female	1.239	1.029	0.992
Age	≤ 35 year	Ref.	Ref.	Ref.
	36–45 year	1.593***	1.274	1.272
	≥ 46 year	1.125***	1.362*	1.365*
Education	Lower or middle education	Ref.	Ref.	Ref.
	Higher education	1.026	1.164	1.161
Training frequency	≤ 1x/week		Ref.	Ref.
	2x/week		1.868***	1.836***
	≥ 3x/week		3.745***	3.604***
Organizational context	Individual		Ref.	Ref.
	Friends, colleagues, small groups		1.021	1.067
	Clubs		1.538**	1.594**
Main sport	Main sport		Ref.	Ref.
	Not as a main sport		0.973	0.999
Event participation	1x/year		Ref.	Ref.
	2-4x/year		1.413**	1.368*
	≥5x/year		2.117***	2.005***
Attitudes toward running	Running as a sport that is easy to practice			0.987
	Perceived advantages of running			1.342*
	Individual motives for quitting			1.070
	Social motives for quitting			0.859
Nagelkerke R^2^		0.029	0.154	0.161

* = *p*<0.05

** = *p*<0.01

*** = *p*<0.001

In Model 2, age still determines the use of sports watches. Older runners (46 years or older) are more likely (OR = 1.362, *p*<0.001) than people in their twenties or thirties to use an app. Regarding the running-related variables, the use of sports watches is related to organizational context, training frequency, and event participation. In contrast to the results found for the usage of apps, club runners are more likely (OR = 1.538, *p*<0.001) to use sports watches than individual runners. In contrast to the usage of apps, event participation has a positive effect on the use of sports watches. Indeed, those who run two or more events a year are more likely to use sports watches than runners who partake in only one event a year (2–4 times a year: OR = 1.413, *p*<0.01 / ≥5 times a year: OR = 2.117, *p*<0.001). The training frequency also contributed to the usage of sports watches. Frequent runners (2 times or more a week) are more likely to use sports watches (2 times a week: OR = 1.868, *p*<0.001 / ≥3 times a week: OR = 3.745, *p*<0.001). While “main sport” is a determinant variable for the usage of apps, this variable does not contribute to the usage of sports watches.

In line with the results of the third binary regression model for the uses of apps, the three selected blocks of variables contribute to explain the usage of sports watches. The usage of sports watches is related to age, organizational context, event participation, training frequency, and perceived advantages of running. Runners aged 46 years or older are more likely (OR = 1.365, *p*<0.05) to use sports watches than those aged 35 years or younger. The usage of sports watches is mainly determined by training frequency. Indeed, people who run twice a week (OR = 1.836, *p*<0.001) and people who run three times or more a week (OR = 3.604, *p*<0.001) are more likely to use a sports watch than those who run only once (or even less) per week. In contrast to the usage of apps, club runners (OR = 1.594, *p*<0.01) and runners who participate in more than one event a year are more likely to use a sports watch (2–4 times a year: OR = 1.368, *p*<0.05 / ≥5 times a year: OR = 2.005, *p*<0.001). Table 6 also reveals that runners who perceive advantages of running are more likely to use sports watches (OR = 1.342, *p*<0.05).

### Consumer profiles

On the basis of the results of the binary logistic regression models, it is possible to make estimations about the probability of the usage of apps and sports watches (see [3]). Table 7 shows, for example, that females aged 36- to 45-years-old, with lower or middle educational levels, running individually twice a week, with running not serving as their main sport, participating in a single event a year, and with a higher score on the psychographic variables “perceived advantages of runners”, “individual motives”, and “social motives for quitting” have a high (75%) probability of using an app. On the other hand, male runners, older than 46 years, with a higher education, who run 3 times or more a week in clubs, with running as their main sport, participation in 5 or more events a year, and have high scores on the psychographic variables “running as a sport that is easy to practice” and “perceived advantages of running” have a 10% probability of using an app. Some other examples of consumer profiles are listed in Table 7.

**Table 7:** Probability of event runners’ usage of apps and sports watches for different consumer profiles.

Socio-demographic			Running-Related				Psychographic				Probability	
Gender	Age	Education	Training frequency	Organizational Context	Main sport	Events	Ease practice	Perceived Advantage	Individual Quitting	Social Quitting	Usage of apps	Usage of sports watches
Male	46 years & older	High	3x/w & more	Clubs	Yes	5x/y & more	High	High	Low	Low	0.10	0.76
Male	46 years & older	High	3x/w & more	Clubs	Yes	5x/y & more	Low	High	High	Low	0.13	0.78
Male	46 years & older	High	3x/w & more	Individual	Yes	2-4x/y	Low	High	High	Low	0.26	0.60
Male	36–45 years	High	2x/w	Small group	Yes	5x/y & more	High	High	Low	Low	0.38	0.51
Male	36–45 years	High	2x/w	Individual	Yes	2-4x/y	Low	High	High	Low	0.54	0.41
Female	36–45 years	High	1x/w & less	Small group	No	2-4x/y	Low	Low	High	High	0.59	0.21
Female	36–45 years	High	2x/w	Small group	No	2-4x/y	Low	Low	High	High	0.62	0.32
Male	36–45 years	High	2x/w	Individual	No	1x/y	High	High	Low	Low	0.64	0.32
Female	36–45 years	Low/middle	2x/w	Individual	No	1x/y	Low	High	High	High	0.75	0.28

## Discussion

The aim of this study was to gain insight into the characteristics of event runners who use apps and sports watches, and to identify the determinants factors of event runners’ usage of apps and sports watches. Gaining insight into which runners use apps and sports watches is key to support runners in healthy and sustainable running (contributing to a decrease in drop-out rates in running) and to adequately address runners in their capacities as consumers. The literature showed that expenditures on wearable monitoring devices are rising in the sporting goods market. Monitoring devices have a considerable and growing share in the total expenditures on running [8,63]. The results in this paper confirm the use of monitoring devices in running; 86% of the participants in the selected half marathon had reportedly used at least one or two monitoring devices over the past 12 months. Results show that about 60% of the respondents use a sports watch. More than half of the respondents reported the use of apps (53.3%). When these results are combined, there is also a considerable group (27.0%) that use both apps and sports watches, while 14% of the event runners use no wearables at all. The brand-specific analysis reveals that the most popular app among runners is Runkeeper, followed by Runtastic and Nike+ Running. Statistics from different app stores are in line with these findings [23,24]. Garmin was found to be the most popular brand among users of sports watches, whereas Polar, TomTom, and Nike are used less.

The second purpose of this study was to identify the determining factors (i.e. socio-demographics, sports-related, and psychographic characteristics) of runners using these monitoring devices. From the findings of the bivariate analyses, significant results were found for gender, age, training frequency, organizational context, main sport, and event participation among app users. For sports watch users, significant differences were found for the variables age, training frequency, organizational context, main sport, and event participation.

Binary logistic regression analysis revealed that there was no consistent relationship between age and usage of apps or sports watches. Table 5 shows that the usage of apps is negatively related to age, which is, given the direct relationship between expenditure on sporting goods and usage of sporting goods, in line with findings from Lamb et al. [39]. Conversely, the usage of sports watches tends to be positively related to age, which is in line with the results of other studies [40,64]. These results indicate that the direction of the relationship between age and expenditure/usage of wearables depends on the type of monitoring device. Possible explanations can be found in smartphone usage in daily life. Younger adults are likely more often early adopters of new technologies [65], which can be a reason why older adults are less likely to use an app to monitor their running.

Male runners do not use monitoring devices more often than female runners. Results showed no significant differences in gender for both types of monitoring devices, while in other studies, gender is a determinant variable in sports expenditure [39,41]. A possible explanation can be found in the sample used in this study in which the distribution of male and female is skewed (resp. 77.4% and 22.6%).

No significant relationships were found between education and usage of monitoring devices. Other studies [36,42] find that groups with lower educational levels spend lower amounts of money on sporting goods, and on overall sports participation. However, the composition of the samples in these studies is different when compared to our study. For instance Lera-López and Rapún-Gárate [36] use a sample with sports participants in general, in contrast to our running specific sample. With regards to education, our sample was comparable to other running samples in previous large-scale running studies in Western Europe, being highly educated (for an overview of these studies, see [1]).

As observed in other studies [47–49], sports-related variables such as training frequency, organization context of running, running as a main sport, and event participation were used to indicate levels of involvement, which seems to be a determinant factor in expenditure on sporting goods. Results indicate that the variable “running as a main sport” has a significant effect on the usage of apps, but no relationships were found for sports watches. Runners for whom running is not their main sport are more likely to use apps. Training frequency and event participation are both positively related, while no significant relationship was found between the training frequency and app-usage, and significant negative relationships were found between app-usage and event participation. McGehee et al. [50] found that training frequency, event participation, and expenditure on running-related products and services increased in individuals with high levels of involvement. Additional results of the variables organizational context of running and running as a ‘main sport’ indicate that there are relationships between these variables and the probability to use monitoring devices, but the relationship depends on the type of device used. For instance, club runners are significantly more likely to use sports watches than individual runners, while club runners are less likely to use apps. A possible explanation can be the social influence in sports clubs. Previous studies have shown that social influence has a significant effect on consumption [66,67]. For example, the purchase of a sports watch by a fellow club member makes it more likely that another sports club member will purchase the same brand and model within a reasonable amount of time. Thus, the sports clubs and their members can often be seen as a more conservative and traditional sector [68]. Therefore, they are more likely to use a more traditional form of monitoring device, such as a sports watch.

The above results indicate that sports-related variables are predictors for the usage of monitoring devices [31,32,46]. It seems that beginners and less involved runners are more likely to use an app, while more experienced and higher involved runners are more likely to use a sports watch.

Psychographic variables (the consumer’s state of mind) give information about AIO’s guiding consumer behavior [46,52]. Examples used in research [49,53–58,69] are consumer’s personality, lifestyle, values, attitudes, beliefs, motivations, and needs. These studies revealed a relation between psychographic variables and the amount of money and time that is spent on sports. In our study app-use is positively related to individual motives for quitting, which means that runners who score higher on this scale (i.e., more likely to stop running based on individual reasons) are more likely to use an app. This is in line with previous research [8], which found that runners who were more likely to quit running, are often novice runners who have less expenditure on sporting goods. Therefore, the use of an app would be more likely than a more expensive device such as a sports watch. One out of four motives contribute significantly to the probability to use a sports watch. In this case, the scale on perceived advantages of running is positively related.

Results indicate that socio-demographics, sport-related variables, and psychographic variables determine the use of apps and sports watches. This is in line with the findings of Ogles and Masters [51] and Scheerder et al. [3], who found that different types of runners were distinguishable not only by their demographic characteristics, but also by their behavioral and psychographic variables. Nevertheless, differences in the nature of the relationships between these variables are dependent on the type of monitoring device that is used.

Apps are more likely to be used by younger, less experienced and involved runners. Therefore, apps have the potential to target this group of novice and fragile runners, who run mostly individually without professional guidance, and are more likely to drop-out from running due to personal reasons. However, these apps require a more personalized and differentiated approaches to target these runners [15]. While more older and more experienced runners with higher involvement, are more likely to use sports watches. This group of runners are more likely involved in clubs with professional guidance. Therefore, they should be targeted differently than novice runners.

Like Scheerder et al. [3] did, the probability of using apps and sports watches for different consumer profiles was estimated on the basis of the results of the logistic regression models. We included all independent variables (both significant and not significant) to give designers and manufacturers of electronic running-related devices a complete view of all variables in these consumer profiles.

Some limitations and questions for further research can be highlighted. As stated in the literature review, income is sometimes found statistically positively related to the decision to take part in sports or not [70] and plays a significant role in the decision to spend money on sports and the amount of money that is spent. However, income was not included as a variable in this study. Next, this study does not allow for making empirically grounded statements for all runners. For this study, a sample of event runners was selected. This is an interesting target group because running events can be considered as a carrier of the growing popularity of recreational and unorganized running [6,10,47,71], although future research could consider different samples to fully reach all potentially different types of runners. In this sample, we only included runners of the half marathon (21.1k) because of the heterogeneity of the participants, including both experienced and less experienced runners. Thus, the results of this study are based on a Dutch sample. Furthermore, some methodological limitations concerning the dependent variables should be mentioned. First, we did not control for the intensity of the use of monitoring devices. Second, we did not consider the differences in the purchase price between apps and sports watches. As mentioned before, a smartphone is a non-specific sporting good that becomes a sporting good when a sports-related app is installed and used. Some app users may consider purchase of the smartphone, while others only count the download of the app. Moreover, the decision-making processes that lead to the use of monitoring devices were not included in this study. Challenges for future research concern further investigation of the popularity and reach of monitoring devices, the underlying motives to use either a sports watch or an app, and a broader focus on participants in 5-10k distance events and non-event runners. Future research should also consider replicating the study in different countries, because sports cultures and the consumption of sporting goods may vary.

## Conclusion

In recent years, there has been an exponential increase in the availability and use of sports and health-related apps, activity trackers, and sports watches. The sporting goods industry has embraced technology in developing products that can motivate and coach people to become and remain active. The findings in this study provide a better understanding of runners’ determinants of running-related apps and sports watch usage. From the results of the logistic regression models, it is possible to estimate the probability of using apps and sports watches for different runner profiles. The constructed consumer profiles provide a tool for designers and manufacturers of electronic running-related devices to better target runners through personalized and differentiated approaches. Segmentation considered socio-demographics, sports-related characteristics, and psychographic variables, which seem to have effectively differentiated between app users and sports watch users.

Apps are more likely to be used by younger, less experienced and less involved unorganized runners. Hence, apps have the potential to target this group of novice and fragile runners. In contrast, sports watches are more likely to be used by a different group of runners, older and more experienced, organized runners with higher involvement. Although apps and sports watches may potentially promote and stimulate sports participation, these electronic devices do require a more differentiated approach to target specific needs of (unorganized) runners. Considerable efforts in terms of personalization and tailoring have to be made to develop the full potential of these electronic devices as drivers for healthy and sustainable sports participation.

## Supporting information

S1 File Dataset ERS2014. Sub-dataset Eindhoven Running Survey 2014 used in this study.
(XLSX)
